# Mixture and Non-Mixture Cure Models for the survival analysis of SARS-CoV-2 patients in Khyber Pakhtunkhwa, Pakistan

**DOI:** 10.12669/pjms.40.8.8931

**Published:** 2024-09

**Authors:** Naseem Asghar, Umair Khalil, Iftikhar Uddin

**Affiliations:** 1 Naseem Asghar, Lecturer, Department of Statistics, Abdul Wali Khan University Mardan, Pakistan; 2 Umair Khalil, Associate Professor, Department of Statistics, Abdul Wali Khan University Mardan, Pakistan; 3 Iftikhar Uddin, Assistant Professor, Department of Community Medicine, Bacha Khan Medical College Mardan, Pakistan

**Keywords:** Cox proportional hazards model, Kaplan-Meier curve, Mixture cure model, non-mixture cure model, SARS-CoV-2

## Abstract

**Objective:**

To examine the potential difference in survival and risk of death between asymptomatic and symptomatic SARS-CoV-2 patients, controlled by age and gender for all the attendance in hospitals of Khyber Pakhtunkhwa (KP), Pakistan.

**Methods:**

In this retrospective study, the medical records of 6273 SARS-CoV-2 patients admitted to almost all hospitals in Khyber Pakhtunkhwa during the first wave of the coronavirus outbreak from March to June 2020 were analysed. The effects of gender, age, and being symptomatic on the survival of SARS-CoV-2 patients were assessed using cure-survival models as opposed to the conventional Cox proportional hazards model.

**Results:**

The prevalence of initially symptomatic patients was 55.8%, and the overall mortality rate was 11.8%. The fitted cure-survival models suggest that age affects the probability of death (incidence) but not the short-term survival time of patients (latency); symptomatic patients have a higher risk of death than their asymptomatic counterparts, but the survival time of symptomatic patients is longer on average; gender has no significant effect on the probability of death and survival time.

**Conclusion:**

The available data and statistical results suggest that asymptomatic and young patients are generally less susceptible to initial infection with SARS-CoV-2 and therefore have a lower risk of death. Our regression models show that uncured asymptomatic patients generally have poorer short-term survival than their uncured symptomatic counterparts. The association between gender and survival outcome was not significant.

## INTRODUCTION

Due to regular global pandemics, healthcare professionals are subject to more stress and anxiety levels due to their direct involvement with patients.^1^ Certain necessary measures can help in control of pandemics.^2^ Most recently, the SARS-CoV-2 infection was declared a global pandemic. By direct contact, inhaling respiratory droplets, or airborne aerosols from the infected patient, the novel SARS-CoV-2 infection spread quickly throughout the world. Since infectious diseases have killed more people than wars in history, therefore, SARS-CoV-2 poses a serious threat to human health.^3^

At first, SARS-CoV-2 cases were classified into four categories: mild, moderate, severe, and critical. However, there is mounting evidence that many SARS-CoV-2 infections are asymptomatic but spread the virus to others. As a result, patients without symptoms are just as contagious as those with symptoms.^4^ Based on a scoping review of the literature, there is significant heterogeneity in estimates of relative infectiousness between asymptomatic and symptomatic SARS-CoV-2 infected individuals. This underscores the need for additional research on this crucial parameter.^5^ Clinical symptoms are the primary criterion used in the population’s infection screening process. Research on the features of clinical symptoms in various populations, particularly in cases that are imported, is, nevertheless, scarce. Research indicates that nonpharmaceutical public health measures, such as face mask laws and social distancing, were crucial in keeping SARS-CoV-2 under control.^6^,^7^

Since the onset of the epidemic, numerous researchers with different expertise tried their level best to understand the dynamics and control of this epidemic. It has been observed that the survival time of patients varies highly and is based on various factors like the underlying health condition and age etc. In standard survival analysis, one of the major assumptions is that all subjects will experience the event of interest once in their lifetime. However, it is often the case that a non-negligible fraction of subjects will never experience the event, and these subjects are referred to as cured having infinite survival times. For instance, some cancer patients will certainly be relapse-free after receiving a certain treatment at which all cancer cells are eliminated. Another example illustrated in this paper is that not all SAR-CoV-2 patients died due to the infection. Standard survival analysis typically conflates the speed of progressing to an event with the cured subjects. To address this problem, cure models were proposed to serve as an extension to the classical survival models.

In the literature, there are mainly two types of cure models for the analysis of time-to-event data with a non-negligible proportion of cured subjects, namely the mixture^8^-^10^ and non-mixture cure models.^11^-^14^ Specifically, let p be the incidence probability where *0 < p < 1*, or equivalently, (*1 – p*) is the cure probability or the proportion of ‘’long-term survivors’’ in the population. Moreover, let *Su(t)* a proper survival function that represents the latency component of a cure model than the overall survival probability at time *t* for a mixture cure model is given by:

*S(t) = (1 – p) + pSu(t). (1)*

The mixture cure model classifies patients into cured and non-cured groups according to the incidence probability *p*. For those who are cured, their survival probabilities at any particular time point are equal to one, whereas, for those who are not cured (i.e., susceptible patients), their survival rates at time t can be described through *Su(t)*. In contrast with the conventional Cox proportional hazards (PH) model which contains only the latency component, the mixture cure model assumes that the effects of the covariates on the incidence of the event and survival times of susceptible patients are different. The cure model is useful in describing a situation where treatment is effective in curing a disease, but the treatment may not be effective in delaying the death time of the non-cured patients, and vice versa. In some complex situations, the effects of a covariate on the incidence and survival can be opposite, say treatment can enhance the chance of being cured, but if it fails to cure a patient, his/her survival can deteriorate. Typically, the covariates can be incorporated into (1) through modelling *p* by the logistic regression model and *Su(t)* the Weibull or Cox PH models. Therefore, the exponent of the covariate effects can still be interpreted as the odds ratio and hazard ratio for the incidence probability and survival of the susceptible patients, respectively. Alternatively, the non-mixture cure model has been proposed to generalize the Cox PH model by having the cumulative hazard function bounded to accommodate a cure proportion, also known as the “bounded cumulative hazard model”.^10^ The overall survival function of a non-mixture cure model may be specified as:

*S(t) = (1 – p)^1-Su(t)^. (2)*

Both survival functions in (1) and (2) go to the cure probability (*1 – p*) as *Su(t)* goes to zero when t is very large. A common choice for the incidence probability *p* in (2) is the complementary log-log model because the exponent of the covariate effects can then be interpreted as the hazard ratio as in the Cox PH model. Although the notation *Su(t)* looks familiar to that described in (1), it does not represent the survival function of a susceptible subject here as no mixing population is assumed, but it could rather be interpreted as a latency component for short-term survival in general. The non-mixture cure model has a biologically meaningful interpretation in some specific contexts. For instance, it can be used to model the underlying number of metastasis-competent tumour cells (with no metastasis in all cells for being cured) and their progression rates in a cancer patient.^11^,^12^

In the presence of censoring especially in right censoring at the end of the follow-up period, the cure model (mixture and non-mixture) is the appropriate model than a standard survival model used by many researchers in cancer studies.^15^,^16^ Although the cure rate of SARS-CoV-2 was high, the rapid transmission of the virus led to a high incidence rate and consequently many deaths. Therefore, the identification of factors associated with the cure or death of SARS-CoV-2 patients was so essential. However, more accurate identification of these determinants depends on the selection of the appropriate model. The Cox model has been used to simulate the survival of patients infected with SARS-CoV-2. Despite being the most widely used model in the field of survival analysis, the high rate of recovery for SARS-CoV-2 patients suggests using a mixture cure model, which can take cure fraction into account.

The survival of SARS-CoV-2-infected patients in Khyber Pakhtunkhwa (KP) has not yet been modelled using the cure model and using the Cox PH model for the analysis of SARS-CoV-2 will mean that every one of the populations will die due to the SAR-CoV-2 which is not the case. Further, it is observed that a large proportion of patients did recover and survive in the long-term, regarded as risk-free from the initial infection, thus the Cox PH model may not be applied reasonably. Keeping in view these two main research gaps, in this work, the authors examine the potential effects of the risk factors, namely gender, sex, and symptomatic indicators on both the death (incidence) probability and the time from illness onset to death of the SARS-CoV-2 patients in Khyber Pakhtunkhwa (KP), Pakistan during the first wave of coronavirus outbreak. More precisely, the study proposes the application of the mixture and non-mixture cure models for the survival analysis of patients suffering from SARS-CoV-2.

## METHODS

### Data Source

In this retrospective study, the dataset was acquired from the office of the Directorate General Health Services, KP, Pakistan, which includes almost all hospital attendance records reported from all districts of the KP to the Directorate during the first wave of SARS-CoV-2 infection, i.e., from March 2020 to June 2020.

Patients in self-isolation were not included. The admission criteria are consistent with the predetermined criteria by the government of Pakistan under the guidelines of WHO. There were 6602 SARS-CoV-2-positive patients in total. The available individual-specific information includes age, gender, date of illness, dates of admission and discharge (or date of expiry), and whether a patient was symptomatic or asymptomatic at the time of diagnosis. In the statistical analysis, we only include the patients aged below 100, and without missing covariates. Eventually, the dataset contains 6273 patients.

### Ethical Approval

The study protocol received ethical approval from the Chairman Ethical Committee Bacha Khan Medical College Mardan, KP (ref: 255(a)/BKMC dated 05/09/2021).

### Cure Model

**Fig.1:**
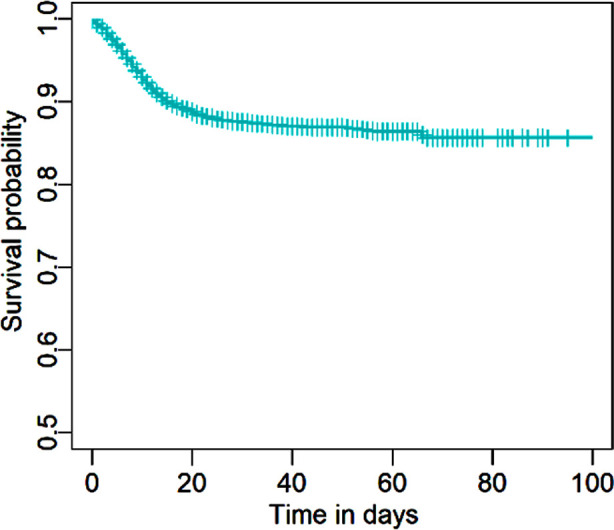
Kaplan-Meier curve for the survival probability based on the KP data.

To check the survival time of SARS-CoV-2 patients during the pandemic, we tried to introduce cure models to check the short and long-term survival of admitted SARS-CoV-2 patients. To apply the model more effectively, we have taken into consideration the patients from different age groups, genders, and health conditions. The primary outcome variable is the time from illness onset to death of the SARS-CoV-2 patients in KP. In Fig.1, we plot the Kaplan-Meier curve for the survival probability based on the data, one can observe a plateau at the right tail. Hence, a significant proportion (i.e., approximately 90%) of patients did recover and were cured from the disease at the date of discharge. It shows that the conventional Cox PH model, which assumes that the survival probability will eventually go to zero (i.e., all individuals would die from the disease given a significantly long period), may not fit the data well. This motivated us to consider the mixture cure model in (1) and the non-mixture cure model in (2). Specifically, we use the logistic regression model and the Weibull regression model for *p* and *Su(t)* in (1). We adopt the complementary log-log and the Weibull regression model for *p* and *Su(t)* in (2). Statistical results with p-values less than 0.05 were considered significant. All computations are performed based on the statistical software R (R for Windows, V4.0.2). The R codes for implementing the mixture and non-mixture cure models are openly available at GitHub https://github.com/lcyjames/WeibullCMs.

## RESULTS

A total of 6273 SARS-CoV-2 positive patients were admitted to hospitals at KP within the study period, among which 4647 (74.1%) were males and 1626 (25.9%) were females; 3498 (55.8%) were asymptomatic and 2775 (44.2%) were symptomatic. The distributions of deaths and recoveries stratified by age are provided in Table-I.

**Table-I:** Number of deaths and recoveries stratified by age at admission.

Age Group	Recovered	Deceased	Row Total
0-9	108	0	108
10-19	254	4	258
20-29	1178	12	1190
30-39	1396	31	1427
40-49	999	99	1098
50-59	854	219	1073
60-69	508	209	717
70-79	192	123	315
80-89	40	37	77
90-99	5	5	10
Column Total	5534	739	6273

A total of 5534 (88.2%) patients recovered with the average length of stay being 30 days, and 739 (11.8%) patients died with the average time from illness onset to death being 10 days. The descriptive statistics of the patients stratified by the symptomatic indicator are given in Table-II. An interesting finding is that, on average, the length of stay of the symptomatic patients was longer, but the time from onset to death was also longer. The numbers of admissions from March to June were 150, 1269,3000, and 1868, respectively, with the corresponding numbers of deaths in these four groups of admissions being 12, 149, 301, and 279.

**Table-II:** Descriptive statistics by symptomatic indicator.

Categories		Asymptomatic (n = 3498)		Symptomatic (n = 2775)	

		Count/Average	Row %	Count/Average	Row %
Age					
≤65	(n = 5742)	3289	57.3	2453	42.7
>65	(n = 531)	209	40.6	322	59.4
Gender					
Male	(n = 4647)	2578	55.5	2069	44.5
Female	(n = 1626)	920	56.6	706	43.4
Outcome					
Recovered	(n = 5534)	3415	61.7	2119	38.3
Length of stay		29.7 days	-	32.2 days	-
Deceased	(n = 739)	83	11.2	656	88.8
Time from onset to death		4.86 days	-	11.2 days	-

We stratify the sample into two age groups, namely those aged at or below 65 and those aged above 65. We study the association between time from illness onset to death and the three covariates: age, gender and being asymptomatic. We first perform a standard survival analysis and fit the Cox model to the data, and the results including the estimated hazard ratios (HR), 95% confidence interval (CI) and -values are provided in Table-III. One can see that age and symptomatic variables are significantly associated (-values < 0.001) with survival of the SARS-CoV-2 patients. As suggested by HRs that are greater than one, old aged and symptomatic patients have an increased risk of death. The association between gender and the survival of patients is shown to be insignificant.

**Table-III:** Results based on the Cox proportional hazards model.

Component	Variable	Estimated HR	95% CI	-Value
Latency	Age65	3.640	(3.091, 4.287)	0.001
	Female	1.025	(0.868, 1.210)	0.769
	Symptomatic	9.634	(7.662, 12.11)	0.001

As illustrated in Fig.1, the outcome of each patient was resolved (either died or recovered) within approximately three months, and presumably, they can be regarded as risk-free from the initial infection after discharge. We then apply the Weibull mixture cure model introduced earlier to the data, allowing a fraction of patients to be cured (risk-free). The results are summarized in Table-IV. In the incidence component, an odds ratio (OR) greater than one indicates an increased probability of being uncured, whereas in the latency component, an HR greater than one represents an increased risk of death given an uncured subject. The results suggest that old aged and symptomatic patients faced a higher probability of being uncured and that an uncured symptomatic patient generally survives longer than those uncured asymptomatic patients.

**Table-IV:** Results based on the Weibull mixture cure model.

Component	Variable	Estimated OR	95% CI	-value
Incidence	Age65	5.656	(4.424, 7.232)	0.001
	Female	1.015	(0.818, 1.258)	0.895
	Symptomatic	14.67	(11.45, 18.78)	0.001
Latency	Age65	1.032	(0.858, 1.241)	0.738
	Female	0.945	(0.789, 1.132)	0.538
	Symptomatic	0.276	(0.215, 0.354)	0.001

The results based on the Weibull non-mixture cure model are given in Table-V. For the incidence component, an HR greater than one generally indicates an increased probability of being uncured. Some complications arise in interpreting the HR in the latency component since we cannot relate this to the survival of an uncured patient as in the mixture cure models. In general, an HR greater than one in latency indicates an increased risk of death in short-term survival. Analogous to the above, age and being asymptomatic are significantly associated with long-term survival, while only the symptomatic indicator is significantly associated with the risk of short-term survival. Coinciding with the results based on the Cox PH model, the gender variable is not significant in both incidence and latency components with the mixture and non-mixture cure models.

**Table-V:** Results based on the Weibull non-mixture cure model.

Component	Variable	Estimated HR	95% CI	-value
Incidence	Age65	3.946	(3.297, 4.722)	0.001
	Female	1.052	(0.878, 1.261)	0.581
	Symptomatic	12.08	(9.549, 15.28)	0.001
Latency	Age65	0.816	(0.645, 1.032)	0.089
	Female	0.917	(0.753, 1.118)	0.390
	Symptomatic	0.229	(0.179, 0.294)	0.001

## DISCUSSION

SARS-CoV-2 was one of the leading concerns in global public health. The primary outcome measure in the study of the SAR-CoV-2 patients was often the time from illness onset to death in the hospital set-up. Also, the underlying effects of several explanatory variables (e.g., age, gender) on this primary outcome were of interest. Research work typically adopted the Cox proportional hazards model in the statistical analysis. However, a major assumption of the Cox model is that the patients will eventually die from the disease which does not fit the scenario of SAR-CoV-2 in practice, as most patients did recover and became risk-free from their initial infection. In this paper, we proposed to use the cure survival models as alternatives. By making use of the available data, we examined the effects of age, gender, and symptomatic indicators on the survival of the patients. Our results show that asymptomatic and young patients were generally less vulnerable to the initial SARS-CoV-2 infection, hence with a lower risk of death.

Our findings indicated a higher risk of death among symptomatic patients, despite longer average survival times compared to asymptomatic patients. However, gender was found to have no significant effect on survival outcomes.

Two features of the SAR-CoV-2 infection made it conspicuous. A significant proportion of patients were asymptomatic, and a substantial number of patients recovered from the initial infection and became risk-free. Further details on asymptomatic infections can be found in systematic reviews.^4^,^5^ Based on the second characteristic, we proposed to use the cure-survival model instead of the Cox-PH model. Our statistical results based on the available data in KP showed that asymptomatic SARS-CoV-2 patients had a lower risk of death since their first infection. One study showed that asymptomatic patients had normal clinical indicators and faster viral shedding than symptomatic patients with similar detected viral load.^6^

They reported that more active cellular immune responses and normal liver function would be the driving factors. On the contrary, our statistical models suggest that the short-term survival prospects of asymptomatic patients who were not cured were worse than those of symptomatic patients. This was also reflected in a shorter average survival time in the first group compared to the second group (Table-II). This could be partly explained by the lack of medical attention for asymptomatic patients and the uncertainty that a patient can deteriorate rapidly. Usually, patients with severe symptoms are allocated more attention and medical resources. The situation would be chaotic if the medical system is overwhelmed with a surge of coronavirus infections, which was likely the case in KP during the first wave.

Our results suggest that people who had close contact with infected patients should also be quarantined and closely monitored during the study period. Our analysis also shows that the prognosis of young patients is generally better, which is consistent with the results of two other studies with comparable study periods.^7^, ^17^ No significant gender difference was found in either the latency or incidence component. One study has shown that the detected viral load in asymptomatic and symptomatic patients was similar, which indicates that infected asymptomatic patients have the potential for transmission, especially in the early phase of infection.^18^ From the public health perspective, an important challenge in curtailing the SAR-CoV-2 outbreak was to identify and quarantine the asymptomatic patients. With the absence of clinical symptoms, screening measures are difficult to implement effectively, while the diagnosis and treatment of asymptomatic patients are often delayed due to poor prevention awareness.^6^,^7^

Therefore, this patient group tends to have a higher risk of transmission compared to symptomatic patients. Public health strategies such as social distancing, face mask mandates, and increasing vaccination coverage play an important role in combating the pandemic. Ideally, all SAR-CoV-2 patients should be included in the study to better reflect the survival pattern of infected patients, but this is difficult to achieve in practice. At the time of the SAR-CoV-2 outbreak, guidelines for the medical and nursing management of infected patients were sparse and under development. Different strategies were used to treat different types of patients and classify them according to the severity of their symptoms. For example, self-isolation at home was recommended for mild symptoms; moderate cases were treated at home or in the hospital, while the more severe cases were admitted to the intensive care unit.

Asymptomatic infection is not initially recognized as one of the categories. This type of biased sampling problem may lead to an over-estimation of the actual death rate as comparatively more severe cases were admitted by the hospitals, as well as an underestimation of the number of confirmed cases in KP, especially in the very early period with a shortage of testing kits and limited medical resources. Moreover, the number of deaths may have been underreported due to the challenges in ascertaining and attributing the causes of death.

## CONCLUSION

In contrast to the conventional Cox-PH model, we have introduced the mixed and non-mixed cure models in this paper. The study provides an evidence-based approach to investigating the short- and long-term survival of SAR-CoV-2 patients. The results of this study may not be generalizable to the later pandemic situation, as the data were collected during the first wave of the coronavirus outbreak in Pakistan when there were no mutant variants of SARS-CoV-2 and no vaccine was available. The probability of death may be overestimated as the self-isolated patients were excluded from the study.

During the first wave of infection, no vaccine was available and there were no mutant variants of SAR-CoV-2, reflecting the basic survival pattern of patients. Further research considering all waves of infection should be conducted to account for these two important factors that are confounding factors in the statistical analysis. The interaction between the presence of symptoms and vaccination is of great importance.

The study focused primarily on gender, age, and symptomatic status as predictors of survival. Although these factors are undoubtedly crucial, other relevant variables such as comorbidities, socioeconomic status, and access to healthcare should be considered in a more comprehensive analysis in the future.

### Authors Contribution:

**NA:** Manuscript draft preparation and statistical analysis and responsible for accountability of study.

**UK:** Methodology and final approval.

**IU:** Data collection and final approval.

## References

[1] Pathiraja PDM, Srikanthi WS, Jayamanne BDW, DeSilva HS (2022). Depression, Anxiety, and Stress among nursing officers in a dedicated hospital for COVID patients in Sri Lanka:A Single Institute Experience. Pak J Med Sci.

[2] Hamid MAB, Tariq S (2021). Challenges of COVID-19 Vaccination Delivery in Pakistan. Pak J Med Sci.

[3] Ali S, Noreen S, Farooq I, Bugshan A, Vohra F (2020). Risk Assessment of Healthcare Workers at the Frontline against COVID-19. Pak J Med Sci.

[4] Gao Z, Xu Y, Sun C, Wang X, Guo Y, Qiu S (2021). A systematic review of asymptomatic infections with COVID-19. J Microbiol Immunol Infect.

[5] McEvoy D, McAloon C, Collins A, Hunt K, Butler F, Byrne A (2021). Relative infectiousness of asymptomatic SARS-CoV-2 infected persons compared with symptomatic individuals:a rapid scoping review. BMJ Open.

[6] Han H, Xu Z, Cheng X, Zhong Y, Yuan L, Wang F (2020). Descriptive, retrospective study of the clinical characteristics of asymptomatic COVID-19 patients. mSphere.

[7] Mei X, Zhang Y, Zhu H, Ling Y, Zou Y, Zhang Z (2020). Observations about symptomatic and asymptomatic infections of 494 patients with COVID-19 in Shanghai, China. Am J Infect Control.

[8] Maller RA, Zhou S (1992). Estimating the proportion of immunes in a censored sample. Biometrika.

[9] Lam KF, Fong DYT, Tang OT (2005). Estimating the proportion of cured patients in a censored sample. Stat Med.

[10] Lam KF, Hongqi X (2005). A Semiparametric Regression Cure Model with Current Status Data. Biometrika.

[11] Omer ME, Bakar MR, Adam MB, Mustafa MS (2020). Cure Models with Exponentiated Weibull Exponential Distribution for the Analysis of Melanoma Patients. Mathematics.

[12] Chen MH, Ibrahim JG, Sinha D (1999). A new Bayesian model for survival data with a surviving fraction. J Am Stat Assoc.

[13] Tsodikov AD, Ibrahim JG, Yakovlev AY (2003). Estimating cure rates from survival data:an alternative to two-component mixture models. J Am Stat Assoc.

[14] Lam KF, Lee CY, Wong KY (2021). Marginal analysis of current status data with informative cluster size using a class of semiparametric transformation cure models. Stat Med.

[15] López CA, Jácome MA, Keilegom IV, Cao R (2020). Nonparametric covariate hypothesis tests for the cure rate in mixture cure models. Stat Med.

[16] Kutal DH, Qian L (2018). A Non-Mixture Cure Model for Right-Censored Data with Fréchet Distribution. Stats.

[17] Park HC, Kim DH, Cho A, Kim J, Yun KS, Kim J (2021). Clinical outcomes of initially asymptomatic patients with COVID-19:A Korean nationwide cohort study. Ann Med.

[18] Zou L, Ruan F, Huang M, Liang L, Huang H, Hong Z (2020). SARS-CoV-2 viral load in upper respiratory specimens of infected patients. N Engl J Med.

